# Confronting racism in family planning: a critical ethnography of Roma health mediation

**DOI:** 10.1080/09688080.2019.1571324

**Published:** 2019-02-15

**Authors:** Charlotte Kühlbrandt

**Affiliations:** Research Associate, School of Population Health & Environmental Sciences, King’s College London, London, UK

**Keywords:** Roma health mediation, family planning, contraception, intersectionality, discrimination, reproductive health, racism, Romania, ethnography

## Abstract

Roma health mediators are part of a government funded, community-led health intervention. One of the programme’s central aims is to improve access to reproductive care for Roma women, often said to be one of the most disadvantaged population groups in Europe. This paper is a critical analysis of mediation in Romania, focusing on how social determinants shape access to family planning and how mediators are employed to address inequalities. It is based on ethnographic observations of mediators at work, as well as in-depth interviews with community members, health professionals, and mediators. Health professionals tended to see Roma families as wanting and having an unreasonably large number of children and tried to curtail this through the promotion of contraception. This contrasted with the perspective of community members, who appeared not to choose having many children but who instead struggled to access contraception for financial reasons. Roma health mediators generally seemed aware of multiple and intersecting pressures that women were facing, but ultimately tended to frame family planning as a matter of choice, culture, and knowledge. I set these perspectives against the background of anti-Roma racism and eugenic sentiments, reflected in popular discourses about Roma reproduction. I explore how an intervention that nominally aims to promote the emancipation of Roma communities, in fact entrenches some of the racially fused assumptions that are connected to inequalities of access to reproductive health care in the first place. The discussion has implications for Roma reproductive health interventions across Europe, and for participatory interventions more globally.

## Résumé

Les médiateurs sanitaires pour les Roms s’inscrivent dans le cadre d’une intervention de santé communautaire financée par les pouvoirs publics. L’un des objectifs centraux du programme est d’élargir l’accès des femmes roms, souvent considérées comme l’un des groupes de population les plus défavorisés en Europe, aux soins reproductifs. Cet article est une analyse critique de la médiation en Roumanie. Il se centre sur la façon dont les déterminants sociaux influencent l’accès à la planification familiale et comment les médiateurs sont employés pour corriger les inégalités. Il est fondé sur des observations ethnographiques des médiateurs au travail, ainsi que des entretiens approfondis avec des membres de la communauté, des professionnels de santé et des médiateurs. Les professionnels de santé tendaient à considérer que les familles roms n’étaient pas à la hauteur et qu’elles avaient un nombre déraisonnable d’enfants. Ils essayaient de remédier à cette situation en encourageant la contraception. Cette opinion contrastait avec la perspective des membres de la communauté, qui semblaient ne pas décider d’avoir beaucoup d’enfants, mais qui avaient plutôt du mal à accéder à la contraception pour des raisons financières. Les médiateurs sanitaires pour les Roms paraissaient dans l’ensemble conscients des pressions multiples et interconnectées que subissaient les femmes, mais ils considéraient en fin de compte la planification familiale comme une question de choix, de culture et de connaissances. Je place ces perspectives dans le contexte du racisme anti-Roms et des sentiments eugéniques, reflétés dans les discours populaires sur la reproduction des Roms. J’étudie comment une intervention qui vise nommément à promouvoir l’émancipation des communautés roms alimente en fait certaines suppositions racistes qui sont en premier lieu liées aux inégalités d’accès aux soins de santé reproductive. La discussion a des conséquences sur les interventions de santé reproductive pour les Roms dans l’ensemble de l’Europe et plus généralement sur les interventions participatives.

## Resumen

Mediadoras Sanitarias Romaníes son parte de una intervención sanitaria dirigida por la comunidad y financiada por el gobierno. Uno de los principales objetivos del programa es mejorar el acceso a los servicios de salud reproductiva para las mujeres romaníes, a menudo consideradas como uno de los grupos más desfavorecidos de la población europea. Este artículo, un análisis crítico de la mediación en Rumania, se enfoca en cómo los determinantes sociales definen el acceso a la planificación familiar y cómo las mediadoras son empleadas para abordar las desigualdades. Se basa en observaciones etnográficas de mediadoras en el trabajo, así como en entrevistas a profundidad con integrantes de la comunidad, profesionales de salud y mediadoras. Los profesionales de salud tendían a pensar que las familias romaníes querían y tenían un número excesivamente grande de hijos, e intentaban restringir este número con la promoción de anticoncepción. Esto contrastaba con la perspectiva de los integrantes de la comunidad, quienes parecían no optar por tener muchos hijos sino que tenían dificultad accediendo a métodos anticonceptivos por razones financieras. Por lo general, las mediadoras sanitarias romaníes parecían ser conscientes de las múltiples presiones interrelacionadas que enfrentaban las mujeres, pero en última instancia tendían a plantear la planificación familiar como cuestión de elección, cultura y conocimientos. Presento estas perspectivas en el contexto de racismo antiromaní y sentimientos eugenésicos, reflejados en los discursos populares sobre reproducción romaní. Exploro cómo una intervención que nominalmente tiene como objetivo promover la emancipación de las comunidades romaníes, de hecho se atrinchera en algunos de los supuestos fusionados racialmente que, en primer lugar, están conectados con desigualdades de acceso a los servicios de salud reproductiva. La discusión tiene implicaciones para las intervenciones en salud reproductiva de romaníes en toda Europa, y para intervenciones participativas a nivel mundial.

## Introduction

Dr Florian, a specialist of obstetrics and gynaecology working in a large municipal hospital, drew me a pie chart with which he tried to demonstrate that the Roma population would soon overtake the Romanian population in terms of its size. He told me that he was concerned about an increased birth rate among the Roma population and how this would affect the demographic fabric of Romanian society. *“I think in fifty years from now they will become the majority here in Romania.” – “And how do you imagine that society to be?”* I asked him. *“A jungle”* he laughed, *“a jungle!”* He elaborated: *“if there are three, four or five million, they can’t be integrated. They start shifting the integration, we as the majority, we will have to start integrating with them.”* Dr Florian’s language was objectifying. The way he spoke was reminiscent of how someone might talk about a threatening epidemic. He said their number was *“growing fast”*, that *“a critical point”* had been reached. He bluntly portrayed the Roma population as a problem for Romanian society; undesirably deviant and Other. His proposed solution was to stop paying families child allowance after their third child, and to further promote free contraception. This narrative of fear, the open anxiety of a shifting demographic pattern is widespread in Romania, and has been well documented and analysed.^1–3^ The public health-focused literature on Roma further consolidates a picture of unequal access to contraception between Roma and non-Roma women, and emphasises gender-based discrimination from within communities, as well as geographic, educational, and financial barriers.^4,5^ Both in the popular, and racially tinged discourse of a “demographic shift”, and in the public health literature, reproduction of Roma women is seen as a “problem”. The Roma health mediation programme is one of the only interventions that have been developed to tackle discrimination and health inequalities faced by Roma women.^6^ It is a state-financed community-led health intervention that employs women of Romani origin to act as a link between Roma communities, health professionals and local authorities. One of its main aims is to address unequal access to contraception. Although the programme has gained much recognition in the field of Roma rights, it has not been the subject of critical in-depth analysis.

## Roma health mediation as a community intervention for family planning services

The Roma are consistently reported as being Europe’s largest and one of its most socio-economically deprived minorities, described as a vulnerable and marginalised group.^7^ The academic and policy literature continues to draw a clear distinction between Roma and non-Roma, even though they struggle to find methods to disentangle what are in effect fluid boundaries between culturally and socio-economically heterogenous groups. The lack of consensus over who counts and who does not count as “Roma” is reflected in the vastly varying headcounts per country. In Romania, for example, which has a total population of just under 20 million, the official census records under 620,000 Romani individuals, in contrast to an NGO estimate of 2.2 million,^8^ while the most commonly cited unofficial estimate is 1.5 million, or 6.7% of the total population.^9^ Much of the literature on Romanian Roma rights and health emphasises how the past five centuries have been marked by cultural and political oppression of Roma, interspersed with violent periods that have left deep scars on the relationship between Roma and non-Roma groups. Romani women, in particular, are portrayed as an archetypal “underserved” population because they are said to face not only ethnic, but also gender-based discrimination from within communities.^10^ Unequal access to reproductive health services is only one part of a wider discourse about Roma as being in need of support and development.^11^ Across Europe, health outcomes are reported to be worse for Roma than for non-Roma.^12–14^ These outcomes have been linked to health-related behaviour, geographic, financial, and educational barriers.^15,16^ Negative experiences of health care include direct and indirect discrimination such as refusal of assistance by health professionals and emergency services, segregation in hospitals, and degrading or inferior treatment.^17^ Health and health-seeking behaviours in Roma communities are often seen as connected to Romani culture,^15,18,19^ even though there has been very little research on the subject, and what little literature exists is of poor quality.^20,21^ The literature on Roma health tends to focus on a narrow set of “problems” of which reproductive health and access to contraception is a recurring theme. What is worrying about this is that the particular gaze offered by the academic and policy literature on Roma health reinforces itself: when a large part of the literature focuses on reproductive health, this topic is likely to be seen as an “objective” problem in Roma communities. At the same time, very few authors seem to be asking Roma communities (however defined) what they see as their own health priorities.

Over the last ten years, more than twenty European member states have trained and employed mediators focusing on health, education and employment.^22^ The Romanian Roma health mediation programme is the first of this kind, based on a small grass-roots initiative, piloted in the 1990s. The Roma health mediation programme can be viewed as a cultural intervention, as it is built on the assumption that Romani women are better able to communicate with other Romani women.^23^ In 2011 there were roughly 380 health mediators working throughout Romania, each nominally serving a community of 500–750 people, though in practice this number is often higher.^23^ A more accurate or up-to-date number of health mediators working in Romania has not been officially published, but the employment of new mediators has stagnated since the programme’s decentralisation after Romania’s financial crisis in 2008–2009. Nevertheless, it is estimated that since its foundation the programme has provided support to between a quarter and a third of the Roma population in Romania.^24^ Because of high rates of abortions and maternal mortality in Roma communities, family planning has been justified as one of the main focus points of the health mediation programme in Romania.^24,25^ Roma health mediators are expected, as is written in the training manual, to respect Romani traditions.^25^ According to their training, their role is explicitly not to force contraception onto Roma communities, but instead to inform, communicate, educate and guide Romani women by discussing advantages and disadvantages of certain forms of contraception and encourage women to choose for themselves with the guidance of a physician.^25^

In Romanian Roma communities, women are exposed to several competing accounts of how reproduction should or should not be regulated. A prominent nationalist Romanian discourse, raised by Dr Florian, claims Roma “should have fewer children”;^24^ conversely, Roma leaders have called for Roma families to continue having children as a defensive strategy to preserve Romani cultural heritage.^2^ Tensions are said to arise particularly between religious demands and cultural traditions that frown upon the use of contraception on the one hand, and poor living conditions on the other hand that often necessitate women restricting the size of their family according to their ability to provide for their children.^2^ Contraceptive methods such as the pill that used to be available free of charge are no longer accessible in an increasingly impoverished Romanian health system.^26^ The uptake of contraception in Roma communities is therefore not merely governed by cultural factors: it has also been hampered by the financial burden that it entails, such that abortion often remains the most accessible form of birth control.^26^

A number of questions arise from the narrative prominence of contraceptive uptake in Roma communities as a “problem”, and the subsequent development of a programme that tries to address this “problem”. To what extent is health mediation able to cater to the needs of the women it serves, and to what extent does it entrench harmful, even racists assumptions (such as those raised by Dr Florian at the outset of this article) about “Romani culture” being the prime factor that discourages contraceptive uptake? This paper uses ethnographic and interview data collected during a year of fieldwork to explore how intersectional factors shape and limit Romani women’s access and use of family planning services and to consider how Roma health mediation tackles existing inequalities between Roma and non-Roma access to contraception. I argue that Roma health mediators do not have the capacity to pay adequate attention to intersectional barriers to contraceptive uptake. I consider some of the unintended consequences of health mediation that arise as a consequence.

## Methods: A critical ethnography of Roma health mediation

This paper is a critical analysis of discursive and enacted forms of mediation, based on rich ethnographic observations of mediators at work, as well as in-depth interviews with community members, health professionals, and mediators. I conducted 11 months of fieldwork in Romania (2014–2015). During this time, I spent two months each with health mediators in two different core case sites, one city and one village. I also visited other mediators for shorter periods to explore a variety of contexts in which health mediators worked. In each site I conducted participant observation and in-depth interviews. The study included three focus group discussions with health mediators and 40 interviews: 13 with health mediators, six with community members, 11 with health professionals (seven doctors and four nurses), and ten with other key informants. Everyone I interviewed gave written consent. In each interview, I followed a topic guide that was tailored to the participant. As I collected them, the interviews were transcribed and translated by a Romanian researcher (Alina Huzui). I coded early interviews line by line, writing analytical notes as I went along.^27^ Open coding led to a coding framework, which helped me to identify common themes. I wrote up my findings with the help of my fieldnotes. All material has been anonymised and I use pseudonyms for the names of places and individuals throughout the paper. Attributes of both people and places have been altered where disclosing them could compromise anonymity. The study received ethical approval from the London School of Hygiene and Tropical Medicine, and local approval from the Romanian Institute for Research on National Minorities.

Throughout the research process, I reflected on my own role in the production of knowledge, the impact of my position on people’s utterances, relationships and behaviours. Conducting research in severely economically deprived communities requires sensitive, contextual, and relational processes of negotiation between the researcher and participants.^28^ In my relationships with key informants, as well as other participants, I was aware of my own position as a non-Roma, non-Romanian young academic, which linked to questions about the legitimacy of critiquing a Roma-led health intervention.^29^ The way that my position as a researcher was constructed gave me a high status as a producer of knowledge, making my research a potentially valuable product for interested parties. I am aware of how my assertions may be instrumentalised in a way that could have implications for those dependent on the programme for employment. However, this study should not be read as an “objective assessment”, but as a product of highly situated, ethnographically produced knowledge. I intend it to act as a contribution to a much-needed constructive debate about how the Roma health mediation and similar programmes may be modified to operate in ways that benefit those individuals and communities that they seek to strengthen.

## Findings

Like Dr Florian, the health professionals I spoke to tended to see Roma families as wanting and having an unreasonably large number of children, a phenomenon that needed to be curtailed. This contrasted with the perspective of community members, who appeared not to choose having many children but who instead struggled to access contraception for financial reasons. Roma health mediators generally seemed aware of multiple and intersecting pressures that women were facing, but ultimately tended to speak about family planning as a matter of choice, culture, and knowledge.

### Health professionals' perspectives

Health professionals framed women’s reproductive decisions as a question of knowledge, individual choice, cultural practice, or a combination of the above. They did not see a financial problem, since they argued that contraception was provided to women for free. “*Roma families have more children in comparison to the Romanian families who don’t have [as many] children. One child, or two children at most. But the others have five or six […] Even if somebody suggested some contraception methods in order not to, they don’t accept them,*” one doctor told me. Doctors’ understanding of the issue included the assumption that Romani women were under cultural pressure to conceive. But they also said that Romani women did not care about contraception, implying a recklessness not found in non-Romani women. Dr Radu told me she thought Roma women had children so that they could then live off the child benefits. These benefits used to amount to as little as €10/month, although now the sum had “doubled” to €18. She emphasised that she had already done everything in her power to increase the uptake of contraception among teenage girls, but they simply would not listen.

During fieldwork I heard comments from another health professional who, referencing Hitler, espoused outright neo-facist views. While I do not wish to imply that this kind of discourse was lurking behind the comments made by other health professionals, the extreme positions that I did encounter suffice to demonstrate how dangerously close these discourses are to an undisguised eugenic desire to govern and police the fertility and reproduction of Roma communities. It is against this background, and against the historical examples of enacted eugenics during the Holocaust and forced sterilisation during and after the Second World War,^30^ that any policy regarding Romani women’s contraceptive choices must be understood. A politics of population containment may be outrightly fascist and eugenicist, but it is important to be equally alert to it when it takes subtler forms; on occasion it may be disguised in the language of progressive liberalism.

### Women’s experiences of contraception

Health professionals’ cultural reasoning as to why there was low contraceptive uptake among women did not on the whole resonate with the conversations I had with women about past, present and future family planning decisions. I spoke to many women, across different parts of Romania, who told me that the expense of contraception prevented them from using it. In some cases, this was more a matter of being informed about ways of accessing free contraception, rather than free contraception being unavailable *per se*. In Romania, family planning is nominally included under a package of free treatment, for both insured and uninsured patients.^31^ In practice, however, treatment is often subject to informal payments.^26^ When women referred to the cost of contraception in conversation with me, it was not always clear whether they were referring to official or informal charges. Sonia in Pădurea, for example, was now taking the pill. She told me that her GP had tried to charge her for contraception, and if she had known earlier that she could get it free from the family planning clinic, she would have been able to prevent her last pregnancy. Another woman said she had only had her last child because a local government agency had stopped giving her three-monthly injections.

The recurrent theme of fertile women was part of everyday life. While I was shadowing a GP, I saw how patients and nurses teased a woman called Adela in the waiting room. She was in her mid-thirties and had seven children. *“How many more children do you want? Will you ever stop?”* they taunted her. Her reply came quick, and with a twinkle in her eye, as though she was well used to the gibes: *“I won’t stop till I’ve had 14, that’s a good number.”* She knew how to self-ironise the dominant discourse about Roma fertility, and used it in her defence. When I spoke to her at her house, with one child breastfeeding on her arm, and the others whizzing every which way and demanding her attention every few minutes, she told me that she did not, in fact, want more children. The two of us were crouching on a log behind her house, out of earshot from her partner, about whom she had few favourable things to say. She thanked the Lord for giving her so many children, because she loved them all dearly, but she had never intended to have this many. She had trouble doing all the washing by hand, she had a painful leg, and she could no longer move freely. The whole family shared a single room, but it was not her house she lived in; it was her partner’s. *“I cannot leave him,”* she whispered to me, *“because I have nowhere to go.”* She said that when she gave birth to her sixth child, she had started taking contraceptive pills, but one month she had not been able to afford them, and she had gotten pregnant immediately. Prior to that, she had gone for three-monthly injections at the GP for four years, during which she had not fallen pregnant once. But then they stopped offering the injections for free, and so she had another child. Now, she said, she wanted to get the coil, but she struggled to save up the money: 250 lei for the coil, 10 lei for the trip, and 300 lei for the cost of the examination: almost 600 lei (£120), she calculated. She said it was not feasible for her at the moment, since whenever she got her hands on 50 lei, she would buy food for the family. But, she sighed, she could also not afford to have another child; she could barely manage the seven she already had. For now, Adela said, she would continue with the pills, even though they made her feel dizzy.

These women were not choosing or planning to have as many children as they did, and in many cases they fell pregnant despite their intention not to have any more children. This was not because they lacked knowledge about different forms of contraception: on the contrary, they knew what was on offer, but perceived it to be financially unaffordable.

### Mediating contraception

Mediators saw their role as gently *persuading* Romani women to take contraception. *“The community has difficulties accepting contraceptive methods, there’s a lot of work to be done […] it’s sensitive,”* Flavia said in one of the focus groups. The other mediators in the group nodded in agreement. Regarding contraception, mediators were in a difficult position, especially in light of nefarious historical precedents around sterilisation. In conversations with me, mediators spoke about the multiple and conflicting reproductive pressures on Romani women, often nuanced, and sometimes contradictory. As mediators, they were frequently exposed to the kind of anti-natalist discourses presented above, while, as Romani women, they also knew about the intersecting financial and cultural pressures facing Roma communities. They spoke of the organisational challenges (registering with a GP), as well as the often hidden and prohibitive costs of contraception (paying for a referral for a gynaecological examination). They also mentioned *“traditional Romani women”*, who they said wanted *“to have as many children as possible”*. They told me about the pressures that women experienced from their husbands, and how men were entitled to leave their wives if they did not bear children soon after their wedding. They also spoke of the changing attitudes towards contraception, saying that women now knew that they should not have too many children. Nevertheless, the mediators tended to speak out in favour of contraception, and told me that they saw it as their responsibility to encourage women to use it.

From the programme’s perspective they were under strict instructions not to give direct advice on contraceptive methods, but rather to refer women to the appropriate medical facility.^25^ Marta told me about information sessions which she organised with a small group of women in the community, answering questions like, *“what am I supposed to do not to get pregnant again?”* or *“what can I do to keep it a secret?”* The mediators in one of the focus groups said they discussed the advantages and disadvantages of different types of contraception. On occasion, they said, they would accompany women to family planning services, *“because they don’t know how to get there”*, and would sometimes even pay the cost of women’s transport. Mediators had different ways of promoting contraception. Some did so by presenting themselves as a positive example of family planning practices, while others told me how they intervened more directly, even by distributing contraception to women. Still others devised elaborate strategies through which they hoped they could influence family planning decisions. Sometimes it was the men who needed more persuasion that the women. Neli, who was part of one of the focus groups, recounted the elaborate story of how she had persuaded a husband that it would be in his favour to allow his wife to use contraception. Other mediators contributed in more direct ways. Silvia told me that she had on occasion personally provided contraception to women in her town. *“The GP gave me the prescribed contraceptive for young mothers […] and I would do the fieldwork of injecting them.”* Silvia said she did this only when women could not go to the GP themselves, because they were *“too busy or didn’t have enough time”*.

Independently of how well intentioned such forms of subtle persuasions or direct distribution were, they cannot be extracted from the generalised climate of continued suspicion towards external interventions in the reproductive decisions of Romani women. I heard reports of recent cases in which patients had accused doctors of unlawfully performing sterilisations without their consent. Viorica, one of the mediators, told me how a number of years ago a woman she knew had officially reported that her daughter had been sterilised against her will. She had come to hospital to give birth, Viorica told me, and had needed a caesarean section. There had been an emergency and a junior doctor had sterilised her without seeking consent from either her or a relative. Viorica told me that she did not want to pursue the complaint as a mediator because she was worried she could lose her job, and because it might have repercussions on the way that she and her family would be treated by hospital staff. Viorica thought that this case of sterilisation was not an isolated case, but part of a larger pattern. A few years ago, there had been a number of cases in which women had had their *“uterine tubes tied without their knowledge”*. She apparently also did not believe that this was simply a medical error. Instead, she tied this incidence to an ethnically targeted anger towards Roma on behalf of medical practitioners. *“It was a difficult period. Practitioners were bitter about gypsies, girls, women, and sterilised them.”* She had wanted to support the women in making a complaint, but she told me that they, too, had been *“afraid of the doctors,”* on whom they and their family may be reliant for future medical treatment.

It is revealing that, with the exception of this case, mediators did not touch upon eugenicist discourses or the historical or contemporary infringements on women’s reproductive rights. Instead, they focused on the emancipatory potential of contraception. They portrayed it as a rung on the developmental ladder upon which Roma communities found themselves, and their responsibility as being to help women to gain more knowledge about and access to forms of contraception. Mediators’ practice of promoting contraception was therefore contiguous with their discourse. But it is still possible that they contributed to the governing of Roma reproduction, even as they benefitted those women whom they helped to acquire contraception.

## Discussion

### Challenging, sustaining and exacerbating reproductive health inequalities

In this paper I have sought to explore the social determinants that shape and limit Romani women’s access and use of family planning services. I have examined the discursive and enacted forms of health mediation, and the ways in which mediators interact with the health system in their attempt to increase uptake of family planning in Roma communities.

Who benefits from health mediation? With regards to contraception, this is a complex and knotted question. It is especially difficult to answer this question, considering that I did not observe mediators enact many of the things they talked to me about. Nevertheless, an analysis of the way in which they spoke about their practice reveals the kind of assumptions that they were based on and gives an indication of how they saw patients and how they believed patients should behave. These expectations were related to broader assumptions about Roma patients as being in need of education and development.

This study confirms reports in the existing literature ^1,4,5^ that point to intersecting barriers for Romani women seeking to exercise their reproductive rights, whether this is to have children or to prevent pregnancy. The discourse of the women I spoke to substantially departed from that of health professionals, whose interest sometimes veered into Malthusian or even openly eugenicist territory. Health professionals tended to emphasise a difference between Roma and non-Roma *culture*, which they believed to be the prime motivating factor for lack of contraceptive uptake. The women I spoke to often had more children than they had planned or wished for, and reported problems accessing family planning services due to perceived financial constraints. Mediators perceived their practices with regard to family planning as being under multiple pressures. They were aware of both cultural and financial constraints, but in their own portrayal of the problem, they tended to approach the promotion of family planning as a cultural rather than a financial issue.

Roma health mediation tries to tackle existing inequalities between Roma and non-Roma access to contraception by increasing knowledge about different contraceptive methods among Roma communities, by gently persuading community members to engage in family planning, and in some cases, even by directly administering contraception. Instead of attending to the needs of communities, mediators spoke out in favour of contraception and saw it as their responsibility to encourage contraceptive uptake. Mediators’ discourse and practice cannot, however, be separated from a climate infused by racial hostility towards Roma communities that was expressed by health professionals, and in discursive and reported enacted attempts at containing reproductive freedom. Most egregiously, there were cases of potentially unlawful medical practice that obstructed Romani women’s fertility against their choice.

Roma health mediation does not challenge the climate of hostility towards Roma communities, nor the simplistic cultural assumptions that fuel it. Instead, the programme is positioned as an intervention that relies on the cultural similarity between mediators and community members, pitting a traditional and “primitive” Roma culture against more developed and “civilised” understandings of family planning in non-Roma culture. This simultaneously obscures socio-economic disadvantage and feeds ideas of cultural homogeneity, as well as that of a simple binary divide between Roma and non-Roma groups that does not account for the fluidity and heterogeneity that exists in Romanian society today. The programme is ill equipped to address the intersectional barriers, particularly those pertaining to financial accessibility.

The proposed solution of health mediation is the promotion of knowledge. This shifts the responsibility for inequalities onto Romani communities themselves, where – judging by the conversations I had with women – lacking access to contraception was less a question of knowledge and the power of persuasion than of financial inaccessibility of appropriate and acceptable methods. Neither health professionals nor mediators acknowledged the salience of this barrier, nor did health mediation contain the mechanisms to address it. One of the consequences that arise from the conflation of cultural and economic barriers is that the blame for inequalities falls on Romani women and their culture, rather than on the health system that does not make contraception freely available to all women. With these tendencies in mind, and despite the frequently beneficial outcomes of health mediators’ work regarding contraception, it is worth questioning the extent to which enacted health mediation was complicit in a project that saw as its aim the policing of Roma reproductive health.

Why might health mediators have stressed cultural over structural factors when talking about contraception in Roma communities? Mediators’ discourse and enacted practice on contraception has to be situated in the structural environment that shapes their power. Mediators may have focused on cultural rather than structural barriers because they felt that this was a domain in which they could more feasibly bring about change. Even though they were aware of multiple and intersecting pressures on women, they may have felt that structural problems relating to the cost and availability of contraception lay outside of their realm of influence, whereas they could influence women’s beliefs and behaviours. At the same time, it should be acknowledged that mediators were heavily restricted by the programme's general mode of operation. Their training focuses on individual-level determinants of accessing health care and proposes individual-level solutions. Mediators tend to be employed on precarious contracts on a minimum wage salary, and often lack institutional support from municipalities or local public health authorities. Mediators also lack a platform through which they might be able to learn from and support each other. In this atomised and uncertain environment, mediators may perceive that they are putting themselves and their jobs at risk if they criticise the health system or individual health professionals, and therefore resort to denouncing the behaviour of individual women instead. Before placing the burden of responsibility for the way health mediation is enacted on the shoulders of mediators, it is therefore important to consider all the ways in which these structural factors shape how mediators interact with the world around them. Mediators were often trying to do their best to help the communities they served whilst finding themselves at a logistic or political impasse.

## Conclusion

Roma health mediation is one of the only interventions that seeks to address the disparities between Roma and non-Roma reproductive health outcomes. In Romania, as in other countries where the programme is active, the mediation programme has been welcomed as helping to overcome known inequalities.^6^ While mediation may help the individuals who participate to obtain treatment, it also de-politicises the structural aspects of the Romanian health system that often exclude Roma individuals from accessing care: for instance, enrolment in the social health insurance system,^32,33^ or discriminatory behaviour by health professionals.^34^ This study goes one step beyond this argument, showing how health mediation not only leaves systemic causes of inequalities unaddressed, but also fails to challenge racist assumptions behind anti-natalist discourses about Roma communities – and worse, how they inadvertently sustain and exacerbate these sentiments by unquestioningly promoting contraception in discourse and practice. Mediators present this not as answering a demand, but instead as a form of education that helps Roma women to become more “civilised”. The programme may not be well placed to address traditional socio-economic inequalities within the country between Roma and non-Roma, but with a shift in focus, it could be used as a vehicle to challenge discourses and practices that fuel structural racism within the state bureaucracy. In order for the programme to benefit community members, health mediators could be encouraged to engage in more politically acute forms of mediation. Realistically, this would require mediators to have secure employment contracts as well as better training and an adequate support network. Training could place more emphasis on the social and economic determinants of accessing health care and encourage mediators to recognise and challenge racist assumptions that underlie dominant discourses about Roma reproduction. A support network for mediators might allow mediators to amplify a more politicised articulation of racism and structural problems in a way that could reach beyond the communities they serve. A more dialogical form of mediation, based on conversation and engagement^34^ rather than encouragement and enforcement, could work towards encouraging communities to recognise the constraints of their own conditions, and to co-design interventions that not only focus on technical solutions to family planning, but also on the wider social, material, and cultural determinants of access to contraception.
